# Correction: Replication of machine learning methods to predict treatment outcome with antidepressant medications in patients with major depressive disorder from STAR*D and CAN-BIND-1

**DOI:** 10.1371/journal.pone.0315844

**Published:** 2024-12-12

**Authors:** John-Jose Nunez, Teyden T. Nguyen, Yihan Zhou, Bo Cao, Raymond T. Ng, Jun Chen, Benicio N. Frey, Roumen Milev, Daniel J. Müller, Susan Rotzinger, Claudio N. Soares, Rudolf Uher, Sidney H. Kennedy, Raymond W. Lam

In the Abstract, the Results section is incorrect. The correct Results section is: Our replicated models predicted TRD in the STAR*D dataset with slightly better balanced accuracy than Nie et al (68%-72% versus 64%-71%, respectively). Prediction performance on our external methodology validation on the CAN-BIND-1 dataset varied depending on outcome; performance was worse for response (best balanced accuracy 6%) compared to remission (7%). Using the smaller set of features found in both datasets improved or did not hinder prediction performance when evaluated on the STAR*D dataset.

In Subject Selection subsection of Methods, there are errors in the paragraphs. The correct paragraphs are: For the cross-validation STAR*D dataset, we re-implemented the subject selection as described in Nie et al [15]. We included subjects who had baseline QIDS-C scores above five, stayed in the study at least four weeks, and either stayed until Level 2 of STAR*D, or left in Level 1 due to achieving remission. After corresponding with the authors of the Nie et al study (S13 Document), we also excluded a small number of subjects who were missing most of their non-QIDS-C data, to better match their dataset.

For our external validation using the CAN-BIND-1 dataset, we adapted the above inclusion criteria. We included subjects with baseline QIDS-SR scores above 5, who stayed until and had QIDS-SR scores from at least four weeks. We again excluded subjects from the STAR*D dataset if they were missing the majority of non-QIDS-C data, or if they were missing baseline QIDS values from the version being used for a model.

In the Training and Evaluation subsection of Methods, there are errors in the second and third paragraph. The correct paragraphs are: We trained models for the STAR*D cross-validation replication using all features, to predict TRD, as defined as failing to achieve a QIDS-C or QIDS-SR score of five or less in after being in the first two levels of the study for at least four weeks in each level. The 20% holdover set was then used to evaluate model performance.

We used separate models trained on the STAR*D data to externally validate performance on the CAN-BIND-1 dataset, using only the overlapping features as previously described. The models were used to predict antidepressant response by eight weeks in the first treatment level, as defined as a 50% or greater reduction in their last QIDS-SR score in this period. We also used them to predict remission by eight weeks, defined similarly as this latest QIDS-SR score being five or less. When training on the STAR*D data, the predicted outcomes were the same, but instead we used the first nine weeks, as STAR*D recorded QIDS scores at week nine instead of at week eight as in the CAN-BIND-1 study.

In the Feature and Subject Selection subsection of Results, there is an error in the last paragraph. The correct paragraph is: For the STAR*D datasets, replicating the subject selection from Nie et al [15] for TRD prediction as defined by QIDS-C criteria results in 218 subjects, with 571 (26.2%) labelled as TRD. These numbers differ slightly from their paper, which reported 2454 subjects with 642 (26.3%) meeting QIDS-C TRD criteria. For the external validation, the STAR*D dataset with QIDS-SR values and overlapping features with the CAN-BIND-1 dataset included 2848 subjects, with 1338 (47.058.6%) achieving a QIDS-SR response by week 9 and 939 (33.0%) achieving remission. The CAN-BIND-1 dataset included 178 subjects, with 62 (34.8%) achieving QIDS-SR response by week 8 and 32 (18.0%) achieving remission. Remission and response rates for other targets are shown in S3 Table.

In the Replication of Cross-Validation subsection of Results, there is an error in the second paragraph. The correct paragraph is: Our models achieved balanced accuracies and AUCs generally numerically higher than those of Nie et al [15]. The highest balanced accuracy was higher in our study compared to Nie et al (72% versus 71%, respectively). Similarly, our highest AUC was higher at 0.79 versus 0.78, respectively. The z-score of Nie et al’s results in our distributions ranges from -4 to -78.

In External Validation subsection of Results, there is an error in the fourth sentence. The correct sentence is: Again, models based on decision trees perform better. Our results are higher for predicting QIDS-SR (AUC 0.76–0.78) remission than predicting response (AUC 0.64–0.69).

In Further Investigations subsection of Results, there are errors in the paragraphs. The correct paragraphs are: To further understand our results, we also compared the performance of response and remission prediction on cross-validation with the STAR*D dataset, as Table 3 shows. We focused on using the Random Forest models without feature selection, given that this was one of the best performing models. Our models continue to predict response worse than they do remission, though the difference is smaller when using QIDS-SR instead of QIDS-CR. The supplementary material documents additional metrics (S10 table), statistical comparison (S11 Table) and feature importance (S12 Table).

We conducted additional cross-validations, again using Random Forests, to investigate whether fewer features could be contributing to the decreased performance of predicting QIDS-SR response external validation (Table 3). On cross-validation, using only the overlapping features between both STAR*D and CAN-BIND-1 has little effect on performance, with balanced accuracy rising or decreasing by less than 1% compared with all STAR*D features. However, we also note that using our feature selection methods to reduce the number of features generally decreases performance compared to using the full features. Elastic net feature selection drops balanced accuracy to 67%, while clustering-χ^2^ lowers it to 65%. Our results for predicting QIDS-SR remission follow a similar pattern on cross-validation (S10 Table), improving when using the overlapping features but not when using feature selection to reduce features. Unlike for QIDS-SR response, QIDS-SR remission results improve when externally validating on CAN-BIND-1, increasing to a balanced accuracy of 72%.

In the Discussion section, there is an error in the fourth sentence of the first paragraph. The correct sentence is: The performance of our replicated prediction is numerically similar, though generally slightly higher, than that achieved by the prior study.

Also, there are errors in the fifth paragraph. The correct paragraphs is: We investigated why response performance dropped on external validation by conducting additional cross-validations on STAR*D datasets. We found that using the 100 features used for the external validation, which are overlapping between STAR*D and CAN-BIND-1 datasets, produced similar performance on both response and remission predictions. This suggests other factors may be leading to the worsened response prediction on external validation, such as inherent differences in patients between the two datasets. It also provides an example where having to use fewer features due to differences between datasets may actually not hamper performance, unlike prior examples–for instance, when Nie et al only used overlapping features on STAR*D cross-validation, they noted performance decreases. This is likely related to the number and types of features; our external validation dataset had more features overlapping than did this prior work; 100 compared to 22.

Then, in the sixth paragraph, the third sentence is incorrect. The correct sentence is: Our results when using the overlapping features has implications for deployment in clinical settings, where it may not be feasible to collect all the information required to replicate all features from a clinical trial, and for further external validation, transfer learning, or other applications where the number of overlapping features between datasets may be limited.

In Tables 3, 4 and 5, the data under Balanced Accuracy and AUC columns are incorrect. Please see the correct version of Tables 3, 4 and 5 here.

**Table 3:** Resulting from replicating a prior study’s cross-validation, predicting treatment-resistant depression according to the Quick Inventory of Depressive Symptomatology, Clinician version (QID-C) scale, using data from Sequenced Treatment Alternatives to Relieve Depression. GBDT: gradient boosting decision tree. Feature selection methods include clustering-χ^2(30 features) and elastic net (31 features). Results reported as Balanced Accuracy and area-under-curve (AUC). As the replicated study only reported one number for their results, we show the z-score of these against the distribution of our results from 100 runs of 10-fold cross-validation. Additional performance metrics and statistics are documented in S4 Table and S5 Table.

	Balanced Accuracy			AUC		
	Our Result	Nie et al study [15]	Z-Score	Our Result	Nie et al study [15]	Z-Score
**Random Forest**						
Full Features	72%	70%	-10	0.79	0.78	-18
Clustering-χ^2^	71%	68%	-10	0.78	0.77	-5
Elastic Net	69%	69%	0.2	0.76	0.76	0.2
**GBDT**						
Full Features	71%	70%	-5	0.78	0.78	-14
Clustering-χ^2^	70%	70%	1	0.77	0.77	1
Elastic Net	70%	70%	-1	0.78	0.76	-6
**XGBOOST**						
Full Features	70%	68%	-9	0.79	0.76	-30
Clustering-χ^2^	70%	67%	-10	0.78	0.73	-38
Elastic Net	69%	68%	-3	0.76	0.76	-1
**L**_**2**_ **Logistic Regression**						
Full Features	69%	64%	-21	0.77	0.69	-78
Clustering-χ^2^	69%	71%	6	0.77	0.73	-23
Elastic Net	70%	71%	4	0.77	0.77	2
**Elastic Net Model**	68%	68%	1	0.75	0.76	4

**Table 4:** Performance of our predictive models when trained on the Sequenced Treatment Alternative to Relieve Depression (STAR*D) dataset, and externally evaluated on the Canadian Biomarker Integration Network in Depression (CAN-BIND-1) trial, predicting both response and remission according to the Quick Inventory of Depressive Symptomatology, Self Report Version (QIDS-SR) scale. See Methods for our definition of these outcomes. No feature selection was used before running the models. Additional performance metrics and statistics are documented in S7 Table and S8 Table. GBDT: gradient boosting decision tree, AUC: area-under-curve.

	QIDS-SR Response		QIDS-SR Remission	
	Balanced Accuracy	AUC	Balanced Accuracy	AUC
**Random Forest**	60%	0.68	72%	0.77
**GBDT**	61%	0.69	73%	0.78
**XGBOOST**	63%	0.69	70%	0.78
**L**_**2**_ **Logistic Regression**	57%	0.64	71%	0.76
**Elastic Net**	64%	0.68	71%	0.76

**Table 5:** Comparison of model performance with different targets and sets of features, using Random Forests. Overlapping features are the 100 features in both Canadian Biomarker Integration Network in Depression’s CAN-BIND-1’s trial and Sequenced Treatment Alternatives to Relieve Depression (STAR*D), while Full uses all 480 features from STAR*D. Clustering-χ^2^ Selection (30 features) and Elastic Net Selection (31 features) refer to using these feature selection techniques as defined in *Methods*. Targets include antidepressant response, remission, or treatment-resistant depression (TRD), as defined in *Methods*. Models trained and evaluated using cross-validation (CV) on STAR*D, and we also report again the results of externally validating models on the CAN-BIND-1 dataset after being trained on STAR*D. We report balanced accuracy and area-under-curve (AUC). Additional performance metrics and statistics are documented in S10 Table and S11 Table. QIDS: Quick Inventory of Depressive Symptomatology, -SR: Self-Report, -C: Clinician.

Evaluation	Target	Features	Balanced Accuracy	AUC
**CV**	QIDS-C TRD	Full	72%	0.79
**CV**	QIDS-SR TRD	Full	69%	0.77
**CV**	QIDS-C Remission	Full	70%	0.77
**CV**	QIDS-SR Remission	Full	69%	0.78
**CV**	QIDS-SR Remission	Overlapping	69%	0.77
**CV**	QIDS-C Response	Full	65%	0.73
**CV**	QIDS-SR Response	Full	65%	0.74
**CV**	QIDS-SR Response	Clustering-χ^2^ Selection	64%	0.71
**CV**	QIDS-SR Response	Elastic Net Selection	64%	0.71
**CV**	QIDS-SR Response	Overlapping	65%	0.73
**External Validation**	QIDS-SR Remission	Overlapping	72%	0.77
**External Validation**	QIDS-SR Response	Overlapping	60%	0.68
