# Supplementary material for: Replication of machine learning methods to predict treatment outcome with antidepressant medications in patients with major depressive disorder from STAR*D and CAN-BIND-1
Source: PLoS One. 2021 Jun 28;16(6):e0253023. doi: 10.1371/journal.pone.0253023 (PMC8238228; doi:10.1371/journal.pone.0253023)
Supplement: S1 Table — (DOCX) [file pone.0253023.s001.docx]

| **CAN-BIND-1** | **STAR*D** |
| --- | --- |
| *Inclusion* | *Inclusion* |
| Outpatients 18-60 years of age | Outpatients 18 – 75 years of age |
| DSM-IV-TR criteria for MDE in MDD, as confirmed by MINI | DSM-IV criteria for single or recurrent nonpsychotic MDD |
| Score ≥24 on the MADRS. | Score ≥14 on the HRSD_17_ |
| Free of psychotropic medications for at least 5 half-lives (i.e. 1 week for most antidepressants, 5 weeks for fluoxetine) before baseline. | Currently not taking CIT or taking for less than 7 days |
|  |  |
| *Exclusion* | *Exclusion* |
| Diagnosis of Bipolar I or Bipolar II disorder. | Diagnosis of Bipolar disorder (I,II not otherwise specified) |
| Any other psychiatric diagnosis that is considered the primary diagnosis. | History of schizophrenia, schizoaffective disorder, psychosis not otherwise specified, anorexia, bulimia, primary obsessive compulsive disorder |
| Substance dependence/abuse in the past 6 months. | Requires immediate hospitalization for substance/alcohol detoxification or treatment |
| Significant neurological disorders, head trauma, or other unstable medical conditions. | Has general medical condition that contraindicates any level 1 or 2 treatment option. Is taking any concomitant medication that contraindicates any level 1 or 2 treatment option |
| Pregnant or breastfeeding. | If female, is pregnant |
| Psychosis in the current episode. | Psychosis in current or past episodes |
| High risk for hypomanic switch (i.e., history of antidepressant- induced hypomania). | Requires antipsychotic medication or mood stabilizers |
| Failed 4 or more adequate pharmacologic interventions (as determined by the ATHF). | Lack of response to an adequate trial of an SSRI in the current episode of MDD |
| Previously failed or showed intolerance to escitalopram or aripiprazole. | History of clear-cut intolerability to, or lack of effect with, an adequate trial of at least one protocol medication in the current episode of MDD |
| Started psychological treatment within the past 3 months with the intent of continuing treatment | Did not respond to 16 or more sessions of cognitive therapy in the current episode of MDD |
| Contraindications to magnetic resonance imaging. | Did not respond to seven or more sessions of electroconvulsive therapy in the current episode of MDD |
| Any significant personality disorder diagnosis (e.g., borderline, antisocial) that might interfere with participation in the protocol, defined by clinician judgment. |  |
| High suicidal risk, defined by clinician judgment. |  |

DSM-IV, Diagnostic and Statistical Manual of Mental Disorders, Fourth Edidtion; -TR, text revision; MDD, major depressive disorder; MDE, major depressive episode; MINI, Mini International Neuropsychiatric Interview; MADRS, Montgomery-Åsberg Depression Rating Scale; ATHF, Antidepressant Treatment History Form; HRSD_17_, Hamilton Rating Scale for Depression 17 items; CIT, citalopram; SSRI, selective-serotonin reuptake inhibitor.
