# Supplementary material for: Replication of machine learning methods to predict treatment outcome with antidepressant medications in patients with major depressive disorder from STAR*D and CAN-BIND-1
Source: PLoS One. 2021 Jun 28;16(6):e0253023. doi: 10.1371/journal.pone.0253023 (PMC8238228; doi:10.1371/journal.pone.0253023)
Supplement: Attachment — Submitted filename: Response to Reviewers.docx [file pone.0253023.s013.docx]

**Response to Editorial and Reviewer Comments**

*Journal Requirements:*

*When submitting your revision, we need you to address these additional requirements.*

*1. Please ensure that your manuscript meets PLOS ONE's style requirements, including those for file naming. The PLOS ONE style templates can be found at*

[*https://journals.plos.org/plosone/s/file?id=wjVg/PLOSOne_formatting_sample_main_body.pdf*](https://journals.plos.org/plosone/s/file?id=wjVg/PLOSOne_formatting_sample_main_body.pdf) *and* [*https://journals.plos.org/plosone/s/file?id=ba62/PLOSOne_formatting_sample_title_authors_affiliations.pdf*](https://journals.plos.org/plosone/s/file?id=ba62/PLOSOne_formatting_sample_title_authors_affiliations.pdf)

**Response:** Confirmed.

*2. Thank you for including your ethics statement: "All participants provided written consent and institutional review boards provided approval. ".*

*Please amend your current ethics statement to include the full name of the ethics committee/institutional review board(s) that approved your specific study.*

*Once you have amended this/these statement(s) in the Methods section of the manuscript, please add the same text to the “Ethics Statement” field of the submission form (via “Edit Submission”).*

*For additional information about PLOS ONE ethical requirements for human subjects research, please refer to http://journals.plos.org/plosone/s/submission-guidelines#loc-human-subjects-research.*

**Response:** We amended the ethics statement in the Methods section and added the same text to the Ethics Statement field of the submission form:

Methods, new text:

All participants provided written consent and institutional review boards at each study site (University of Toronto Health Sciences Research Ethics Board, University of British Columbia Clinical Research Ethics Board, University of Calgary Conjoint Health Research Ethics Board, Queen’s University Health Sciences and Affiliated Teaching Hospitals Research Ethics Board, McMaster University Hamilton Integrated Research Ethics Board) provided ethics approval.

*3. Thank you for stating the following in the Competing Interests section:*

*"JN, TN, YZ, RTN, JC, and RU have no competing interests.*

*BNF has a research grant from Pfizer.*

*RM has received consulting and speaking honoraria from AbbVie, Allergan, Janssen, KYE, Lundbeck, Otsuka, and Sunovion, and research grants from CAN-BIND, CIHR, Janssen, Lallemand, Lundbeck, Nubiyota, OBI and OMHF.*

*SHK has received honoraria or research funds from Abbott, Alkermes, Allergan, BMS, Brain Canada, CIHR, Janssen, Lundbeck, Lundbeck Institute, Ontario Brain Institute, Ontario Research Fund, Otsuka, Pfizer, Servier, Sunovion, Xian-Janssen, and holds stock in Field Trip Health.*

*RWL has received ad hoc speaking/consulting fees or research grants from: Allergan, Asia-Pacific Economic Cooperation, BC Leading Edge Foundation, Canadian Institutes of Health Research (CIHR), Canadian Network for Mood and Anxiety Treatments (CANMAT), Canadian Psychiatric Association, Healthy Minds Canada, Janssen, Lundbeck, Lundbeck Institute, MITACS, Myriad Neuroscience, Ontario Brain Institute, Otsuka, Pfizer, University Health Network Foundation, and VGH-UBCH Foundation. "*

*We note that you received funding from a commercial source: Lundbeck, Bristol-Myers Squibb, Pfizer, and Servier.*

*Please provide an amended Competing Interests Statement that explicitly states this commercial funder, along with any other relevant declarations relating to employment, consultancy, patents, products in development, marketed products, etc.*

*Within this Competing Interests Statement, please confirm that this does not alter your adherence to all PLOS ONE policies on sharing data and materials by including the following statement: "This does not alter our adherence to PLOS ONE policies on sharing data and materials.” (as detailed online in our guide for authors* [*http://journals.plos.org/plosone/s/competing-interests*](http://journals.plos.org/plosone/s/competing-interests)*). If there are restrictions on sharing of data and/or materials, please state these. Please note that we cannot proceed with consideration of your article until this information has been declared.*

**Response:** We have amended the Competing Interests Statement by adding the following text:

CAN-BIND is sponsored by non-for-profit and government funding agencies and by commercial sponsors including Lundbeck, Bristol-Myers Squibb, Pfizer, and Servier. None of the sponsors were involved in study design, conduct of the study, data analysis, data sharing (except for the government-funded Ontario Brain Institute, as stated in the Data Availability Statement), interpretation of results, drafting of the manuscript, or final approval and publication of the manuscript. This does not alter our adherence to PLOS ONE policies on sharing data and materials.

*Please include your amended Competing Interests Statement within your cover letter. We will change the online submission form on your behalf.*

*Please know it is PLOS ONE policy for corresponding authors to declare, on behalf of all authors, all potential competing interests for the purposes of transparency. PLOS defines a competing interest as anything that interferes with, or could reasonably be perceived as interfering with, the full and objective presentation, peer review, editorial decision-making, or publication of research or non-research articles submitted to one of the journals. Competing interests can be financial or non-financial, professional, or personal. Competing interests can arise in relationship to an organization or another person. Please follow this link to our website for more details on competing interests:* [*http://journals.plos.org/plosone/s/competing-interests*](http://journals.plos.org/plosone/s/competing-interests)

**Response:** Done.

*4. We note that you have indicated that data from this study are available upon request. PLOS only allows data to be available upon request if there are legal or ethical restrictions on sharing data publicly. For information on unacceptable data access restrictions, please see* [*http://journals.plos.org/plosone/s/data-availability#loc-unacceptable-data-access-restrictions*](http://journals.plos.org/plosone/s/data-availability#loc-unacceptable-data-access-restrictions)*.*

*In your revised cover letter, please address the following prompts:*

*(Please add the following in, per reviewer request)*

*The Python 3 code used to prepare the data, and train and evaluate models, is available in a* [*GitHub*](https://github.com/jjnunez11/antidep_pred_replication) *repository, which also contains the raw result files.*

**Response:** Done. Added text:

The Python 3 code used to prepare the data, and train and evaluate models, is available in a [*GitHub*](https://github.com/jjnunez11/antidep_pred_replication) repository , which also contains the raw result files.

*a) If there are ethical or legal restrictions on sharing a de-identified data set, please explain them in detail (e.g., data contain potentially identifying or sensitive patient information) and who has imposed them (e.g., an ethics committee). Please also provide contact information for a data access committee, ethics committee, or other institutional body to which data requests may be sent.*

*b) If there are no restrictions, please upload the minimal anonymized data set necessary to replicate your study findings as either Supporting Information files or to a stable, public repository and provide us with the relevant URLs, DOIs, or accession numbers. Please see* [*http://www.bmj.com/content/340/bmj.c181.long*](http://www.bmj.com/content/340/bmj.c181.long) *for guidelines on how to de-identify and prepare clinical data for publication. For a list of acceptable repositories, please see* [*http://journals.plos.org/plosone/s/data-availability#loc-recommended-repositories*](http://journals.plos.org/plosone/s/data-availability#loc-recommended-repositories)*.*

*We will update your Data Availability statement on your behalf to reflect the information you provide.*

**Response:** CAN-BIND is funded by the Ontario Brain Institute, which requires that all deidentified study data must be securely stored and publicly available through Brain-CODE (<https://braininstitute.ca/research-data-sharing/brain-code>). The research ethics boards also require that deidentified study data be available only through Brain-CODE. We have now included this statement in the Data Availability section.

- Data Availability, new text:

The raw CAN-BIND-1 data is available from Brain-CODE, based at the Ontario Brain Institute (<https://braininstitute.ca/research-data-sharing/brain-code>).

*5. One of the noted authors is a group or consortium [CAN-BIND Investigator Team]. In addition to naming the author group, please list the individual authors and affiliations within this group in the acknowledgments section of your manuscript. Please also indicate clearly a lead author for this group along with a contact email address.*

*[Note: HTML markup is below. Please do not edit.]*

**Response:** Because CAN-BIND is a large consortium, we propose to remove the group from the author list, and instead provide an acknowledgment, with a link to the online listing of investigators.

- The revised text in the Acknowledgments section:

CAN-BIND is an Integrated Discovery Program carried out in partnership with, and financial support from, the Ontario Brain Institute, an independent non-profit corporation, funded partially by the Ontario government. We acknowledge the contributions of the [CAN-BIND Investigator team](https://www.canbind.ca/about-can-bind/our-team/full-can-bind-team/) (https://www.canbind.ca/about-can-bind/our-team/full-can-bind-team/). The opinions, results and conclusions are those of the authors and no endorsement by the Ontario Brain Institute is intended or should be inferred. Additional funding was provided by the Canadian Institutes of Health Research (CIHR), Lundbeck, Bristol-Myers Squibb, Pfizer, and Servier. Funding and/or in kind support is also provided by the investigators’ universities and academic institutions. All study medications are independently purchased at wholesale market values. JJN is partially supported by CIHR Team Grant: GACD Mental Health, GAC-154985.

*Reviewers' comments:*

*Reviewer's Responses to Questions*

*Comments to the Author*

*1. Is the manuscript technically sound, and do the data support the conclusions?*

*The manuscript must describe a technically sound piece of scientific research with data that supports the conclusions. Experiments must have been conducted rigorously, with appropriate controls, replication, and sample sizes. The conclusions must be drawn appropriately based on the data presented.*

*Reviewer #1: Partly*

*Reviewer #2: Yes*

*2. Has the statistical analysis been performed appropriately and rigorously?*

*Reviewer #1: N/A*

*Reviewer #2: Yes*

*3. Have the authors made all data underlying the findings in their manuscript fully available?*

*The PLOS Data policy requires authors to make all data underlying the findings described in their manuscript fully available without restriction, with rare exception (please refer to the Data Availability Statement in the manuscript PDF file). The data should be provided as part of the manuscript or its supporting information, or deposited to a public repository. For example, in addition to summary statistics, the data points behind means, medians and variance measures should be available. If there are restrictions on publicly sharing data—e.g. participant privacy or use of data from a third party—those must be specified.*

*Reviewer #1: No*

*Reviewer #2: Yes*

*4. Is the manuscript presented in an intelligible fashion and written in standard English?*

*PLOS ONE does not copyedit accepted manuscripts, so the language in submitted articles must be clear, correct, and unambiguous. Any typographical or grammatical errors should be corrected at revision, so please note any specific errors here.*

*Reviewer #1: Yes*

*Reviewer #2: Yes*

*5. Review Comments to the Author*

*Please use the space provided to explain your answers to the questions above. You may also include additional comments for the author, including concerns about dual publication, research ethics, or publication ethics. (Please upload your review as an attachment if it exceeds 20,000 characters)*

*Reviewer #1: This paper replicates a machine learning model related to Major Depressive Disorder, gets consistent performance on the same dataset compared to the original study, and evaluates the model on an external dataset. Please see my comments below.*

*The performance dropped by ~10% when applied the model to the external dataset. The study claims that different feature selection methods may improve the performance on the external dataset and presents the results on Table 5. However, this basically means the model is actually tuned on the external dataset, which might make the results biased. By tuning the models and validating on the external dataset multiple times, the external dataset is no longer 'external' anymore. I would suggest the following: (1) split the original dataset into N folds; (2) train the model using the full features using N-1 folds and evaluate on the remaining fold, repeating N times; (3) repeat (2) using different feature selection methods as proposed in the study; (4) comparing the mean and standard deviation between (2) and (3) to firstly show the feature selection improves the performance on the original dataset. If so, apply these models to the external set and compare mean and standard deviation again.*

**Response:** We believe the reviewer brings up valid and important concerns regarding external validation, but they do not apply to our work, which already follows their suggestions and hence does not have these issues. We did not rerun external validation for the results in Table 5, or in general. Table 5 shows the results of cross-validation when changing different parameters in the way the reviewer suggests; the external validation results are the same as shown previously.

We have used this feedback to revise our manuscript to be clearer that we do not break these important tenants of external validation:

- Abstract, new text:

Using the smaller set of features found in both datasets generally improved prediction performance when evaluated on the STAR*D dataset.

- Methods: We added a new subsection heading, ***Further Investigations,*** to separate this section from ***External Validation*** subsection. We also added new text:

We further evaluated models with different combinations of targets (TRD, response, or remission), QIDS scale version (clinician or self-report), feature sets, and subject inclusion criteria, to investigate how these changes affect performance. We evaluated these models through cross-validation on the STAR*D data.

- Table 5, new text:

Models trained and evaluated using cross-validation (CV) on STAR*D, and we also report again the results of externally validating models on the CAN-BIND-1 dataset after being trained on STAR*D.

*Also, please also include specificity, sensitivity, and F1-score. The current two metrics are not enough to show the full spectrum of the performance.*

**Response:** The reviewer also requests that we show important additional metrics and statistics. These were provided in the supplementary material, but we only briefly mentioned this. We have updated each of the result sections and relevant tables to explicitly mention these additional materials. We believe showing these additional statistics in the current tables would make them too crowded but would be happy to add them if preferred.

- Results, new text:

We document sensitivity, specificity, F1 and other performance metrics in S4 Table, statistical comparison between these results in S5 Table, and feature importance in S6 Table.

We again provide further performance metrics (S7 Table), statistical comparison (S8 Table), and feature importance (S9 Table) in the supplementary materials.

The supplementary material documents additional metrics (S10 table), statistical comparison (S11 Table) and feature importance (S12 Table).

- Table 3, new text:

Additional performance metrics and statistics are documented in S4 Table and S5 Table.

- Table 4, new text:

Additional performance metrics and statistics are documented in S7 Table and S8 Table.

- Table 5, new text:

Additional performance metrics and statistics are documented in S10 Table and S11 Table.

*Further, the study should explain in detail why chose this model to replicate. In general, there are many more advanced models can be applied to this problem. Not sure why chose to replicate this specific model instead of developing a novel model.*

**Response:** We have added further justification to our introduction section regarding why we choose this specific replication.

- Introduction, new text:

We are unaware of any prior studies where a set of different authors have replicated work predicting antidepressant treatment outcomes (9,22)

- Introduction, new text:

We chose to replicate the Nie et al study given its relatively strong results, its use of a well-known study that now has its data publicly available, and its evaluation of a variety of common machine learning algorithms.

- Introduction, added text:

An independent external validation is the most rigorous method to establish that results from a predictive model will be valid when applied to a different dataset (23,24), though there are few examples of its use in this field (9,25).

We explained why we believe replication is important in the Introduction section. We agree there are more advanced models that can be applied to this problem, and plan to further explore this in future work, but believe that the models replicated generally perform well and showcase a variety of techniques based on logistic regression and decision trees.

*Reviewer #2: Manuscript Summary:*

*The authors successfully replicated the previous work of Nie et al that predicted antidepressant treatment outcomes using machine learning methods and clinical data. They used a newer version of the STAR*D dataset and a different method for selecting a hold-out set. The results showed slightly better balanced accuracy than that from Nie et al. The external validation on the CAN-BIND-1 dataset and the feature selection were performed, and they claim that the smaller set of features generally improved prediction performance.*

*Some comments:*

*Although this manuscript successfully repeated previous studies and improved the model performances, the innovation of the manuscription is a bit limited. Please provide the significance and more advantages of this project.*

**Response:** We have added additional details to the significance and advantages of this work in the discussion and conclusion sections:

- Discussion, new text:

To our knowledge, this is the first instance of an independent group replicating prior antidepressant outcome prediction, as prior studies have generally published novel, positive results (9,25).

- Discussion, new text:

This may suggest that alternative techniques and parameters for feature selection may lead to increased performance. Whether it be with different feature selection techniques, different models, or other changes, those seeking to improve the performance of predicting antidepressant effect will be able to easily reproduce our work, using the same dataset. This will allow better understanding of how possible changes affect performance.

- Conclusion, new text:

Our results represent the first independent replication of prior work using machine learning models to predict antidepressant outcomes in non-psychotic MDD. We successfully replicate the prior Nie et al cross-validation results, using a newer version of their data that is publicly available. Our external validation of these methods on a new, independent dataset from a different country found that performance was similar to cross-validation when predicting clinical remission, although performance was reduced when predicting clinical response.

*Please consider adding the number of features set based on Clustering-χ2 and elastic net methods in the cross-validation results (table 3), if they are available.*

**Response:** We have added these numbers to Table 3 and to the relevant paragraph in the Methods section:

- Methods, new text:

We again used elastic net optimized to find 31 non-zero weights to select those features.

“…, calculated $\chi^{2}$ scores, and selected the top 30 features based on these…”

- Table 3, new text:

Feature selection methods include clustering-$\chi^{2}$(30 features) and elastic net (31 features)**.**

*AUC is generally used to evaluate the performance of a binary classification model. In this manuscript, please consider adding more description on how to calculate the AUC values in the methods section.*

**Response:** Our work uses AUC to represent binary classification performance. We used functions from scikit-learn’s metrics module when possible to aid replicability, including for the AUC calculation. We have added this detail to the Methods section:

- Methods, new text:

We used functions from scikit-learn’s metrics module to compute specific metrics such as the AUC (40).

*The link about Python 3 code in the GitHub repository should be provided in the Data Availability section.*

**Response:** We have added the requested link to the cover letter.

*Please briefly introduce the differences of STAR*D database between the previous article (Nie et al) and this manuscript.*

**Response:** We agree that further describing differences between the datasets would be beneficial. Unfortunately, due to the Nie et al database being unavailable to us, we are unable to provide any further details to these differences besides what we describe already in the various sections. We have added a sentence to the Methods section to address this:

- Methods, new text:

We were unable to access this older version of the dataset used by the prior authors, though we know there are some differences such as in feature names and formatting (S13 Document).
